# Efficient CsPbBr_3_ Quantum-Dot Light-Emitting Diodes Using Sputtered NiO Films as Hole Injection Layers

**DOI:** 10.3390/ma16176060

**Published:** 2023-09-04

**Authors:** Pao-Hsun Huang, Sih-An Chen, Li-Wei Chao, Jia-Xun Xie, Ching-Yu Liao, Zong-Liang Tseng, Sheng-Hui Chen

**Affiliations:** 1 School of Ocean Information Engineering, Jimei University, Jimei District, Xiamen 361021, China; 201961000111@jmu.edu.cn; 2 Organic Electronics Research Center, Ming Chi University of Technology, No. 84, Gungjuan Rd., New Taipei City 24301, Taiwan; a1010102002@gmail.com (S.-A.C.); aa0932693328@gmail.com (L.-W.C.); u09157151@mail2.mcut.edu.tw (J.-X.X.); m09158020@mail2.mcut.edu.tw (C.-Y.L.); 3 Department of Electronic Engineering, Ming Chi University of Technology, No. 84, Gungjuan Rd., New Taipei City 24301, Taiwan; 4 Department of Optics and Photonics, National Central University, Taoyuan 320317, Taiwan

**Keywords:** CsPbBr_3_ quantum dot, light-emitting diode, NiO, sputter

## Abstract

Perovskite quantum dots (QDs) have showed excellent optoelectronic properties to extend the application range of novel solid-state lighting, such as perovskite QD based LEDs (QD-LEDs). However, the traditional device structure of perovskite QD-LEDs employed PEDOT:PSS as a hole inject layer (HIL), which impairs stability due to acidic surface characteristics. This study proposes the sputtered NiO films as an HIL to replace acidic PEDOT:PSS. The NiO films with significantly different characteristics were prepared by controlling the sputtering parameters to investigate the devices’ performance of NiO-based CsPbBr_3_ QD-LEDs. The optimized device showed an excellent performance with maxima luminescence of 20,118 cd/m^2^ and an external quantum efficiency (EQE) up to 3.63%.

## 1. Introduction

Inorganic perovskite cesium lead halides, CsPbX_3_ (X = I, Br, Cl), have drawn much attention from researchers in the last decade [1]. The photoluminescence (PL) wavelength of cesium lead halides can be adjusted by varying the halide composite (Cl, Br, and I) to cover the whole-visible light spectrum, which is considered as a feasible material for optoelectronic devices. The quantum dots (QDs) of cesium lead halides present more advantages, such as a narrow spectrum, pure color, and high luminescence, to the extent that inorganic perovskite QDs have become one of potential materials for light-emitting diodes (LEDs).

In 2015, the first study on the QD-based LEDs (QD-LEDs) reported on employing inorganic CsPbX3 as emission materials [2], opening a new generation of QD-LEDs. Subsequently, much effort has been dedicated to improve the efficiency of the perovskite QD-LEDs in the aspects of carrier balance and ligand modification [3,4]. Poly(3,4-ethylenedioxythiophene) polystyrene sulfonate (PEDOT:PSS) was usually regarded as a hole inject layer (HIL) for the perovskite QD-LEDs in previous studies due to its high electronic conductivity at room temperature [5]. However, PEDOT:PSS is an acidic material which leads to the erosion of indium tin oxide (ITO). Furthermore, it attributes indium and tin atoms dissociated from the anode to other layers in the devices, resulting in the cause of exciton quenching [6,7] and the reduced stability of the device [8,9]. In addition, the energy barrier between the PEDOT:PSS and QDs is too large to cause charge imbalance [9]. In order to improve the carrier imbalance and chemical instability of PEDOT materials, nickel oxide (NiO) was presented to replace PEDOT:PSS as an HIL and eliminate the above-mentioned disadvantages. NiO is a p-type semiconductor with high conductivity and a wide bandgap with high transparency in the visible light region [8,10]. A higher conduction band of NiO and good valence band matching with the QD layers can effectively transport holes from the QD layers and block the migration of electrons for perovskite solar cells [11]. In addition, quasi-2D perovskite LEDs using NiO as hole injection materials have been reported to control interface defects and improve carrier transporting for efficient devices [12]. Although solution-processed NiO films were chosen to be a replaceable material in the optoelectronic devices [7,9,12,13,14,15,16], the sputtering process for NiO films is more promising and feasible because of its high throughput, mass production, and excellent fabrication stability. However, to the best of our knowledge, no studies regarding the sputtered NiO films for perovskite QD-LEDs were reported.

Herein, the sputtered NiO films were demonstrated for the perovskite CsPbBr_3_ QD-LEDs. Two kinds of NiO thin films with significantly different characteristics, high transparency and low conductivity, and low transparency and high conductivity were prepared by controlling the sputtering parameters. The resultant device with an optimized NiO film achieved an excellent device performance with an external quantum efficiency (EQE) of 3.63% and maxima luminescence of 20,118 cd/m^2^.

## 2. Materials and Methods

### 2.1. Materials

9,9-Bis [4-[(4-ethenylphenyl)methoxy]phenyl]-N2,N7-di-1-naphthalenyl-N2,N7-diphenyl-9H-Fluorene-2,7-diamine (VB-FNPD) were obtained from the Lumtec Corp. (New Taipei, Taiwan). Nickel and nickel oxide targets and an aluminum evaporation slug were bought from the Ultimate Materials Corp. (Hsinchu, Taiwan). Poly(3,4-ethylenedioxythiophene) polystyrene sulfonate (PEDOT:PSS), lead dibromide (PbBr2), zinc acetate dihydrate (Zn(CH_3_COO)_2_·2H_2_O), cesium carbonate (Cs_2_CO_3_), octadecene (ODE), oleic acid (OA), octylamine (Octam), didodecyl dimethyl ammonium bromide (DDAB), tris-[1-phenyl-1H-benzimidazole] (TPBi), lithium fluoride (LiF), toluene, ethyl ethanoate (EtOAc), hexane, and octane were bought from the Uni-Onward Corp. (New Taipei City, Taiwan).

### 2.2. Synthesis of CsPbBr_3_ QDs

Cs_2_CO_3_ (0.032 g), ODE (9 mL), and OA (0.75 mL) were loaded into a glass bottle (50 mL) and then stirred and heated at 120 °C for 30 min to obtain a colorless and transparent cesium oleate precursor solution. PbBr_2_ (0.1104 g), Zn(CH_3_COO)_2_·2H_2_O (0.0186 g), and ODE (10 mL) were loaded into a glass bottle. Then, heat the solution for it to completely dissolve in the heating and stirring machine, and vacuum during the period. Then, inject 0.6 mL OA and 0.6 mL Octam into the solution and heat up to 150 °C for 5min. After that, quickly inject 0.8 mL cesium oleate precursor into the hot solution and cool it in an ice-water bath. Then, DDAB (0.186 g), OA (2.4 mL), and toluene (8 mL) were stirred and heated at 100 °C until they completely dissolved and were injected into the above as-cooled solvent, and after 5 min, were cooled in an ice-water bath to synthesize CsPbBr_3_ quantum dots. The as-synthesized solution was centrifuged at 6000 rpm for 15 min, 15 mL hexane was added to the precipitate (on the bottom) and centrifuged at 6000 rpm for 15 min. The fluorescent green solution in the upper layer was decanted and mixed with EtOAc (at a volume ratio of 1:1). The mixture was centrifuged at 6000 rpm for 15 min and the precipitate was collected. A quantity of 4 mL of octane was added into the precipitate as a dispersing solvent to obtain the CsPbBr_3_ QDs solution.

### 2.3. Device Fabrication

The patterned indium tin oxide (ITO) -coated glass substrates were cleaned by deionized water, acetone, and isopropyl alcohol through an ultrasonic oscillator for 10 min each and the residual cleaning solvents were taken out by pressurized nitrogen. Then, the substrates were treated to UV-ozone for 25 min to improve the surface-work function. There are two kinds of nickel oxide (NiO) thin film sputter in substrates. The two kinds of nickel oxide (NiO) thin film, as a hole inject layer (HIL), were deposited by the DC magnetron sputtering method from different preparation conditions, such as targets, sputtering power, and substrate temperature, respectively. One of them was sputtered from the Ni target with a DC power of 100W and deposited on a room temperature substrate. Another one was sputtered from the NiO target with a DC power of 200 W and was deposited on a substrate at 200 °C. Before both of them, during the sputtering period, the cavity was vacuumed to 2 × 10^−6^ torr and then pure argon and oxygen gas was introduced at the rate of 40 sccm and 100 sccm, respectively. The thickness of the NiO films was controlled by the adjustment of deposition time. After the NiO films were deposited, 9,9-Bis [4-[(4-ethenylphenyl)methoxy]phenyl]-N2,N7-di-1-naphthalenyl-N2,N7-diphenyl-9H-fluorene-2,7-diamine (VB-FNPD), as the hole transporting layer (HTL), was spin-coated at 6000 rpm for 20 s on an HIL, with a roasting temperature of 120 °C for 10 min. Then, the QDs solution was spin-coated at 2000 rpm for 20 s on an HTL in the glove box which was filled with nitrogen. After that, tris-[1-phenyl-1H-benzimidazole] (TPBI), lithium fluoride (LiF), and an aluminum (Al) cathode were deposited into the sample by a thermal evaporator in a vacuum environment. The thickness for TPBI, LiF, and Al was 40, 1, and 100 nm, respectively. The deposition rates for TPBi, LiF, and Al were 0.5~0.6, 0.02~0.03, 0.22~0.24 nm/s, respectively. Finally, the samples were encapsulated with glass substrates using UV glue in a nitrogen environment.

### 2.4. Measurement

The absorbance spectra, photo luminance and the photoluminescence quantum yield of QDs solutions were measured using the fluorescence spectrophotometer (F-7000, Hitachi, Tokyo, Japan) with an excitation wavelength of 365 nm. The TEM image and crystal size distribution were taken on the JEOL JEM-2100 (JEOL Ltd., Tokyo, Japan) and fitted using the origin 8.5 software. The crystal morphology of the QDs were measured using the X-ray diffractometer. The roughness of the films was measured using the atomic force microscope (INNOVA, Bruker, Billerica, MA, USA). The phase of the QDs was confirmed using an X-ray diffractometer (XRD, Rigaku, MiniFlex 600, Akishima-shi, Japan). The transmittance and conductivity of the NiO film was measured using the HALL measurement and the UV-visible spectrophotometer (V-770, JASCO, Tokyo, Japan). The EL spectra, luminance, current density, voltages, and external quantum efficiency of the QDs-LED characteristics were measured using the LQ-100R system (ENLI Technology, Kaohsiung City, Taiwan) with a 100 mm PTFE integrated sphere. The time-resolved PL (TRPL) decay was analyzed using the optical microscope-based system (Fluoromax, Horiba, Kyoto, Japan).

## 3. Results

**Figure 1:**
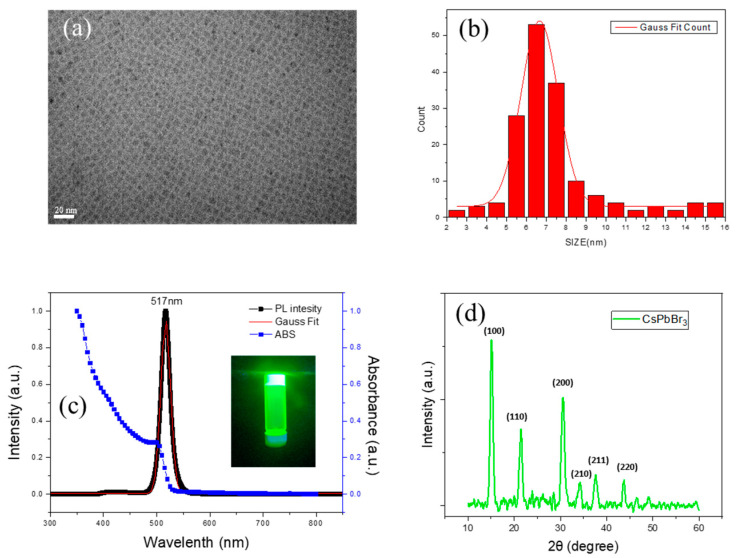
(**a**) High-resolution TEM image, (**b**) particle size distribution, (**c**) PL absorption, and (**d**) XRD pattern of CsPbBr_3_ QDs.

Figure 1a shows a transmission electron microscopy (TEM) image, verifying that CsPbBr_3_ QDs have good uniformity and a cubic shape. The particle size distribution of CsPbBr_3_ QDs exhibits an average size of 6.65 nm with a small standard deviation of 0.02 nm, as shown in Figure 1b. Figure 1c shows the absorption spectrum and photoluminescence (PL) emission band of CsPbBr_3_ QDs, where the green emission peak at 517 nm is close to the absorption edge, which is consistent with previous reports [1,2]. Moreover, the excellent photoluminescence quantum yield (PLQY) of the resulting QDs is up to 95.2%. The X-ray diffraction (XRD) pattern (Figure 1d) identifies (100), (110), (200), (210), (211), and (220) peaks to confirm the traditional cubic structure (JCPDS No. 54–0752) of the CsPbBr_3_ QDs, indicating the reason for a cubic shape in TEM images.

**Figure 2:**
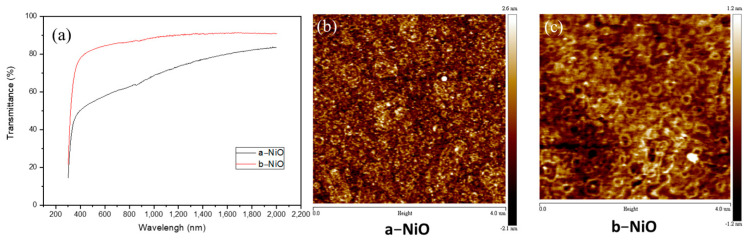
(**a**) Transmittance spectra of both NiO films. AFM images of (**b**) a−NiO and (**c**) b−NiO films.

The NiO films were utilized by the magnetron sputtering deposition method for fabrication onto ITO layers. One of the NiO films (labelled a-NiO) was deposited by sputtering from a metallic nickel target which had non-stoichiometric properties, and is attributed to defects such as the interstitial oxygen and nickel vacancy in the film [11,17]. Moreover, the defects are purposely increased by raising the sputtering power and deposition at room temperature, resulting in an advantage over electrical conductivity. The other one (labelled b-NiO) was deposited using a nickel oxide ceramic target with a lower power and heating treatment under the sputtering process to form high stoichiometric NiO films which had reduced defects and improved crystallinity, indicating higher light transmittance but lower conductivity [18,19]. The transmittance spectra of both NiO films are significantly different, as shown in Figure 2a. The resistivities and transmittances for the a-NiO and the b-NiO at 517 nm are 55.2 and 82.8%, respectively. However, the atomic force microscope (AFM) indicates excellent roughness on both films, as shown in Figure 2b,c, in which root-mean-square roughness (Rqs) is 0.745 and 0.449 nm for the a-NiO and b-NiO, respectively. The process conditions of these two NiO films and their corresponding characteristics are summarized in Table 1.

**Figure 3:**
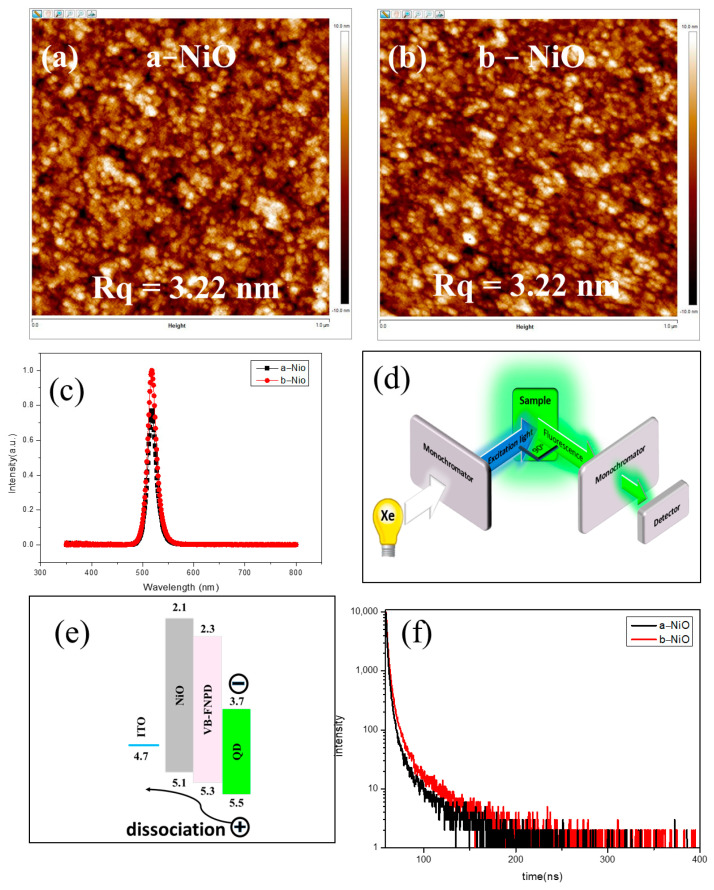
AFM images of (**a**) QDs/VB-FNPD/a−NiO/ITO and (**b**) QDs/VB−FNPD/b−NiO/ITO film stacks. (**c**) PL spectra of (**a**,**b**). (**d**) PL experiment illustration, (**e**) Energy level diagram of QDs/VB−FNPD/ NiO/ITO and (**f**) TRPL spectra of (**a**,**b**) at 517 nm.

After coating VB-FNPD and CsPbBr_3_ QDs on both NiO films (ITO/NiO/VB-FPND/QDs), the surface morphologies and Rqs are almost the same, as shown in Figure 3a,b. Moreover, the thickness of both QD films is also the same (~30 nm), which is confirmed by the anα-step surface profiler. Interestingly, the photoluminescence (PL) behaviors of both NiO films are significantly different, as shown in Figure 3c. The PL intensity of the QD film coated on the a-NiO is obviously weaker than that of the QDs coated on b-NiO. The result of the different PL characteristics is not due to the low transmittance of a-NiO (Figure 2a). The incident light hit the QD films on VB-FNPD/NiO/ITO, and the fluorescence of the QD films directly emitted to the detector in our PL system, as shown in Figure 3d. The energy level diagram of the NiO films is summarized in Figure 3e. The HOMO and LUMO energy levels of ITO, NiO HIL, VB-FNPD and QD layers can be referred to in previous reports [20,21]. The p-type NiO thin films are wide-bandgap semiconductor materials with a HOMO energy level close to VB-FNPD, ensuring a hole transportation from VB-FNPD into NiO. Subsequently, the impact of the QDs/VB-FNPD/NiO interface still requires further discussion before we incorporate this film stack into the device. The time-resolved photoluminescence (TRPL) was performed to confirm the exciton dissociation behavior at the QDs/VB-FNPD/NiO interface, as shown in Figure 3f. Correspondingly, the exciton lifetimes of QDs/VB-FNPD/b-NiO is higher than that of the QDs/VB-FNPD/a-NiO film stack, indicating that the b-NiO film can suppress the exciton quenching from the QD layer to the VB-FNPD. Therefore, the results can be associated with the different resistivity of the NiO films, leading to the higher PL intensity of QDs/VB-FNPD/b-NiO in Figure 3c.

**Figure 4:**
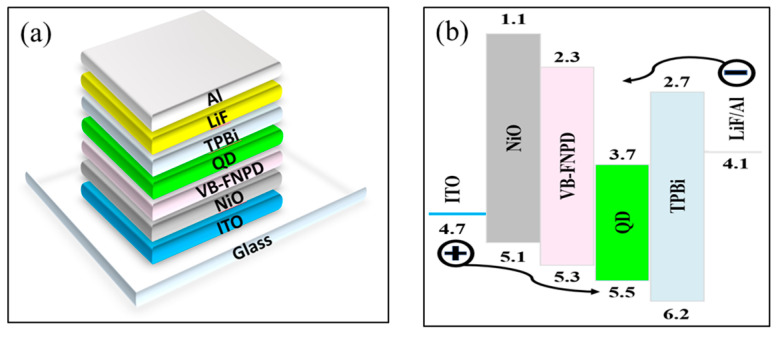

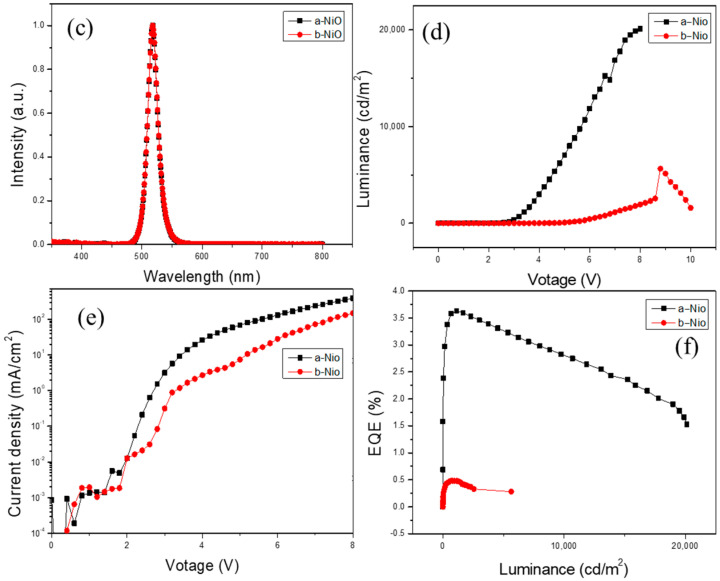
(**a**) Device structure, (**b**) energy level diagram, (**c**) EL spectra, (**d**) luminescence–voltage, (**e**) current density–voltage, (**f**) EQE-luminescence characteristics of NiO-based perovskite QD-LEDs.

Figure 4a shows a schematic of the devices’ structure consisting of a multilayer structure, i.e., ITO/NiO/VB-FNPD/CsPbBr_3_/TPBi/LiF/AL. Figure 4b shows the energy level diagram of the CsPbBr_3_ QD-LED. The EL spectra of both devices (Figure 4c) show almost the same electroluminescence (EL) peak profile at 517 nm with a narrow full-width half-maximum (FWHM) of 21.7 nm, corresponding to the PL emission band of QDs in Figure 2c. Figure 3d shows the luminescence–voltage curves of both devices, where the a-NiO device has a maximum luminance of 20,118 cd/m^2^ at 8.0 V, which is brighter than the b-NIO device (having a maximum luminance of 5637 cd/m^2^ at 8.8 V. Moreover, the turn-on voltage of the a-NiO device (2.2 V) is lower than that of the b-NiO device, which confirms that the device using the a-NiO for HIL is better due to low resistivity. In Figure 4e, the current density of the a-NiO device shows a sharp increase after 2.0 V, and one that is significantly higher than that of the b-NiO device, which may be attributed to the low resistivity. As a result, Figure 3f shows that the maximum external quantum efficiency (EQE) of 3.63%, which is 7.6 times higher than that of the b-NiO device, demonstrating that resistivity is an important factor in the performance of the NiO-based perovskite QD-LEDs.

## 4. Conclusions

We have demonstrated that sputtered NiO films were used as an HIL in perovskite CsPbBr_3_ QD-LEDs to replace traditional PEDOT:PSS HIL. High-quality CsPbBr_3_ perovskite QDs with excellent uniformity and PLQY were prepared to coordinate the sputtered NiO HIL. The a-NiO (high transparency and low conductivity) and the b-NiO (low transparency and high conductivity) were used to discuss the carrier transporting mechanisms of QDs/VB-FNPD/NiO/ITO film stack and the device performance of NiO-based CsPbBr_3_ QD-LEDs. The results exhibit that the resistivity of the NiO films has a high impact in the devices’ efficiency. The optimized a-NiO device achieved a low turn-on voltage of 2.2 V, an external quantum efficiency (EQE) of 3.63%, and a maxima luminescence of 20,118 cd/m^2^.

## Figures and Tables

**Table 1:** Optical and electrical properties of both NiO films.

Sample	Target	Power (W)	Temperature (°C)	Resistivity (ohm/sq)	Transmittance at 517 nm (%)	R_q_ (nm)
a-NiO	Ni	200	Without heating	6.58 × 10^4^	55.2	0.745
b-NiO	NiO	100	controlled at 200 °C	4.46 × 10^6^	82.8	0.449

## Data Availability

The data presented in this study are available on request from the corresponding author.
